# Influence of Experimental Autoimmune Prostatitis on Sexual Function and the Anti-inflammatory Efficacy of Celecoxib in a Rat Model

**DOI:** 10.3389/fimmu.2020.574212

**Published:** 2020-09-09

**Authors:** Yadong Zhang, Xiangping Li, Kuikui Zhou, Mingkuan Zhou, Kai Xia, Yunlong Xu, Xiangzhou Sun, Yingjie Zhu, Chunyan Cui, Chunhua Deng

**Affiliations:** ^1^Department of Urology and Andrology, The First Affiliated Hospital, Sun Yat-sen University, Guangzhou, China; ^2^Shenzhen Key Lab of Drug Addiction, The Brain Cognition and Brain Disease Institute (BCBDI), Shenzhen Institutes of Advanced Technology, Chinese Academy of Sciences, Shenzhen-HongKong Institute of Brain Science-Shenzhen Fundamental Research Institutions, Shenzhen, China; ^3^Imaging and Minimally Invasive Intervention Center, Sun Yat-sen University Cancer Center (SYSUCC), Guangzhou, China

**Keywords:** experimental autoimmune prostatitis, chronic prostatitis/chronic pelvic pain (CP/CPP) syndrome, sexual dysfunction, depression, celecoxib, serotonin

## Abstract

Experimental autoimmune prostatitis (EAP) is a well-established model induced by an autoimmune response to prostate antigen. The symptomatic, pathological, and immunological characteristics of EAP animals are highly consistent with human chronic prostatitis/chronic pelvic pain syndrome (CP/CPPS), which makes EAP an ideal model for this disease. Here, we investigate the influence of EAP on male rat sexual function and the efficacy of anti-inflammatory therapy with celecoxib. EAP rat models were established using male Wistar rats. Rats were randomly assigned to a normal control group, an EAP model group, or an EAP model with celecoxib treatment group (celecoxib group). Behavioral changes, sexual behavioral changes, and erectile function were estimated using an open-field test, a sucrose consumption test, mating experiments, and by intracavernous pressure/mean arterial pressure ratio (ICP/MAP). Histological changes in the prostate were observed by HE staining, and the serum inflammatory factors IL-1β and TNF-α levels were measured by enzyme-linked immunosorbent assay. In addition, serotonin (5-hydroxytryptamine, 5-HT), 5-HT_1A_ receptor, 5-HT_2C_ receptor, and serotonin transporter (SERT) expression levels in the hippocampus and spinal cord (T13–L1, L5–S2) were examined by immunohistochemistry and western blot analysis. Results showed that EAP rats exhibited characteristics of depression, decreased sexual drive, premature ejaculation, and increased threshold of penile erection. Moreover, all these changes were effectively alleviated by celecoxib. Significant increases in prostatic interstitial infiltration by inflammatory cells and in serum IL-1β and TNF-α levels were observed in EAP rats, and these were partially reduced by celecoxib. Additionally, the expression pattern of serotonin system regulators in the hippocampus and spinal cord were altered in EAP model rats, including a decrease in 5-HT levels and an increase in 5-HT_1A_ receptor levels. In conclusion, autoimmune prostatitis impaired rat sexual function, and this was effectively prevented by anti-inflammatory therapy with celecoxib. Moreover, a serotonin system disorder in the central nervous system was likely mediated via inflammation in EAP rats.

## Introduction

Chronic prostatitis/chronic pelvic pain syndrome (CP/CPPS) is an inflammatory disease affecting around 8.2% of the male population throughout the world (1). Common complications of CP/CPPS patients include sexual dysfunction and anxiety-related disorders (2, 3), as confirmed in our previous study (4). In our single-center study, the incidences of premature ejaculation (PE) and erectile dysfunction (ED) in CP/CPPS patients were about 45.3 and 47.4%, respectively (4), while the prevalence of PE was reported up to 64.1 and 36.9% in prostatitis-like symptom and chronic prostatitis group from another epidemiological investigation (5). According to a previous study (6), the incidence of anxiety-related disorders was reported to be 37–60%, including but not limited to depression, anxiety, and trauma-related disorders. Moreover, anxiety-related disorders comprised the highest risk factors for sexual dysfunction in CP/CPPS based on our data (4). However, the relationship among CP/CPPS, anxiety-related disorders, and sexual dysfunction was difficult to clarify because of the various confounding factors.

The cellular and molecular mechanisms underlying CP/CPPS remain largely unknown. Bacterial infection, urine reflux, autoimmune response, neuroendocrine disorder, and anxiety-related factors have been reported to associate with CP/CPPS (7). Based on the theory that immunopathogenic mechanisms (8) are involved, an experimental autoimmune prostatitis (EAP) animal model was established in rodents by generating an autoimmune response to various antigens, including an homogenate of male accessory glands (MAG) (9) or prostate (10), human prostate acid phosphatase (PAP) (11), prostate steroid binding protein (PSPB) (12), and peptide T2 (13). EAP rat is characterized by a prostate-specific cellular and humoral immune response with active inflammatory monocyte infiltration (14). In addition, the rodent model is associated with signs of chronic pelvic pain (15). Since the symptomatic, pathological, and immunological characteristics of the EAP model are highly consistent with human CP/CPPS, EAP rat is an ideal model to investigate effects related to prostate inflammation.

Celecoxib is a non-steroidal anti-inflammatory drug (NSAID) that reduces the production of prostaglandin by specifically inhibiting cyclooxygenase-2 (COX-2), thus producing anti-inflammatory and analgesic effects (16). Several clinical studies have reported that anti-inflammatory therapy with celecoxib alleviated the symptoms of CP/CPPS (17, 18). Also it’s noteworthy that celecoxib was reported to benefit the treatment of depression (19, 20). As an important central neurotransmitter, serotonin (5-HT) is closely related to premature ejaculation and depression (21), which are both manifested in CP/CPPS patients. In clinical practice, selective serotonin reuptake inhibitors (SSRIs) are widely used to treat PE through the inhibition of reabsorption of serotonin in the synaptic gap (22). Thus, the association between inflammation and the serotonin system may be a factor of interest in these investigations.

In the present study, we report that autoimmune prostatitis caused depression-like behavior and impairment of sexual function in the EAP rat model. In addition, the expression patterns of serotonin system regulators in the central nervous system and serum inflammatory factors were altered in EAP rat, and these changes could be prevented by anti-inflammatory therapy with celecoxib.

## Materials and Methods

### Experimental Animals

Specific pathogen-free (SPF) Wistar rats were procured from the experimental animal center of Sun Yat-sen University (Guangzhou, China). Rats were housed in an SPF environment in a standard housing room under controlled temperature (15–25°C), relative humidity (50–70%), and artificial light (12 h light/dark cycle), and were provided free access to food and water. All animal protocols were approved by the ethics committee of The First Affiliated Hospital of Sun Yat-sen University (Guangzhou, China).

### Animal Model and Study Design

Adult male Wistar rats (3 months old, weighing 250–300 g) were randomly divided into EAP model group (*n* = 16), normal control group (*n* = 10), and EAP model with celecoxib treatment group (celecoxib group) (*n* = 10). In addition, prostate protein was extracted from another 18 adult male rats. The EAP rat model was established in the EAP model group and celecoxib group following a modified classical modeling method of two-step immunization (9). Briefly, prostates excised from Wistar rats were homogenized in 0.5% Triton X-100 (Sigma, Aldrich, United States) with an electric homogenizer (HZYBDJ, Hangzhou, China). Prostate protein extract was obtained by centrifugation (12,000 *g*, 30 min) of prostate homogenate and used as antigen. The protein concentration of the supernatant was determined using a BCA Protein Assay Kit (Cwbiotech, Beijing, China), and the supernatant was then diluted to 40 mg/mL. To generate the EAP model, a 1 mL suspension of prostate protein extract (20 mg) and complete Freund’s adjuvant (0.5 mL, Sigma, Aldrich, United States) was injected subcutaneously on day 0, and a 1 mL suspension of prostate protein extract (20 mg) and incomplete Freund’s adjuvant (0.5 mL, Sigma, Aldrich, United States) was injected on day 21 again. In the normal control group, 1 mL of 0.15 M saline was injected subcutaneously. On the day of model establishment, celecoxib (Pfizer Inc., New York, NY, United States) was administered in the celecoxib treatment group by gavage (18 mg/kg/day). In the model group and the normal control group, 0.15 M saline was administered by gavage (0.1 mL/kg/day).

### Open-Field Test

Rats were tested in a self-made square box (80 cm by 80 cm; by 40 cm in height) painted black on the inside. The box bottom was divided into 25 small squares. At the start, the rat was positioned in the central square, and all movements within the next 5 min were recorded by camera. The number of squares that all four limbs of the rat had entered during exploration was taken as a score of horizontal movement. The number of times the rats’ forelegs lifted off the bottom or climbed the wall of the box was taken as a score of vertical movement. The tests were repeated twice by two researchers who were blinded to the grouping of rats, and an average value was taken as the final score. Each rat was tested before model establishment and 8 weeks after.

### Sucrose Consumption Experiment

The Sucrose consumption experiment was performed according to previously published protocols (23). Prior to the start of the experiment, the rats were behaviorally adapted to drinking 1% sucrose water for 2 days. For the first 24 h, two bottles containing 1% sucrose water were placed in each cage. For the second 24 h, one bottle was filled with 1% sucrose water, and the other bottle with pure water. After 24 h water deprivation, each rat was given one bottle filled with 1% sucrose water and one bottle filled with pure water. The total liquid consumption and sucrose water consumption of each rat was determined 1 h later. The sucrose water preference was calculated as follows: Sucrose water preference = [(sucrose water consumption/total liquid consumption) × 100%]. Each rat was tested before model establishment and 8 weeks after.

### Mating Experiment

For mating experiments, 36 adult female Wistar rats (3 months old, weighing 220–240 g) were castrated by bilateral ovariectomy following anesthesia by intraperitoneal injection of pentobarbital sodium (35 mg/kg). Two weeks after the procedure, estrus was induced in castrated female rats at 48 and 4 h prior to mating. Estrus was induced by subcutaneous injection of 20 μg estradiol benzoate (Kingyork, Tianjin, China) and 500 μg progesterone (Kingyork, Tianjin, China) dissolved in 0.1 mL olive oil. The mating experiment was conducted between 19:00 and 21:00. Each male rat was carefully placed into the observation cage for 10 min of adaptation. An estrous female rat was then carefully transferred into the cage. The mating behaviors over 30 min were observed and recorded by camera. The following parameters were evaluated: (i) Mount latency (ML), the time interval between the beginning of the test and the first time that the male rat mounts the female rat, with or without intromission; (ii) Intromission latency (IL), the time interval between the beginning of the test and the first time that the male rat intromits; (iii) Ejaculation latency (EL), the time interval between the first intromission and ejaculation; (iv) Mount Frequency (MF), a count of mount behaviors in 30 min; (v) Intromission frequency (IF), a counts of intromission behaviors in 30 min; (vi) Ejaculation Frequency (EF), a count of ejaculation in 30 min; (vii) Postejaculatory interval (PEI), the time interval between two successive ejaculations.

### Intracavernous Pressure (ICP) and Mean Arterial Pressure (MAP) Measurement

Anesthesia was performed by intraperitoneal injection of pentobarbital sodium (35 mg/kg). The left common carotid artery was exposed by a median cervical incision and a PE-50 catheter connected to a pressure transducer was inserted for continuous MAP measurement. Crus penis and cavernous nerve (CN) were exposed by a lower abdominal incision. A bipolar electrode engaged the right CN with electrical stimulation (20 Hz, 5 V, 0.2 ms pulse duration) for 1 min. A 23G needle connected to a pressure transducer was inserted into either corpus cavernosum through the crus penis for ICP measurement. After 5 min, the electrical stimulation and measurement were repeated. To eliminate the interference of blood pressure, ICP/MAP was adopted to assess erectile function.

### HE Staining of Prostate

After measuring ICP and MAP, the rat’s prostate was harvested, washed with 0.15 M saline, fixed in 4% paraformaldehyde, and embedded in paraffin at 4°C overnight. Serial 4 μm thick sections were stained with hematoxylin and eosin (HE). After HE staining, pathological changes of the prostatic tissue in each group were observed under light microscopy (Olympus, Japan).

### Serum TNF-α and IL-1β Enzyme-Linked Immunosorbent Assay (ELISA)

Blood samples were collected before rat sacrifice and centrifuged (3,000 *g*, 15 min, 4°C) to obtain serum. Serum was stored at −70°C until assayed. TNF-α and IL-1β were analyzed by ELISA kits (R&D Systems, Minneapolis, MN, United States) according to manufacturer’s protocols. Serum TNF-α and IL-1β levels were determined following measurement of absorbance at 450 nm using a Bio-Rad microplate reader (Bio-Rad, Hercules, CA, United States).

### Immunohistochemistry (IHC)

The expression levels of 5-HT, 5-HT_1A_ receptor, 5-HT_2C_ receptor, and SERT in the central nervous system were analyzed by IHC as follows. Brain and spinal cord (T13–L1 and L5–S2) tissues were harvested and fixed with 4% paraformaldehyde, dehydrated with ethanol and graded concentrations of xylene, and finally embedded in paraffin. The paraffinized tissues were then stored in liquid nitrogen until further processing. Sections of 4 μm thickness were blocked by BSA (Santa Cruz, CA, United States) for 1 h at room temperature, and then incubated overnight with primary antibodies against 5-HT (ab10385, Abcam, Cambridge, United Kingdom, 1:200), 5-HT_1A_ receptor (sc-10801, Santa Cruz, CA, United States, 1:200), 5-HT_2C_ receptor (sc-10802, Santa Cruz, CA, United States, 1:200), and SERT (sc-13997, Santa Cruz, CA, United States, 1:200) at 4°C. The sections were then washed and incubated with secondary antibodies and visualized using a DAB kit (K5007, DAKO, Glostrup, Denmark) according to manufacturer’s protocols. Images were obtained using an LSM800 or LSM 710 confocal microscope (Zeiss, Heidenheim, Germany). For quantitative analysis, the results were presented as integrated optical density (IOD) using Image-Pro Plus 6.0 (Media Cybernetics, United States).

### Western Blot

The expression levels of 5-HT, 5-HT_1A_ receptor, 5-HT_2C_ receptor, and SERT in the central nervous system were analyzed by western blot as follows. Brain and spinal cord (T13–L1 and L5–S2) tissues were lysed in 1 × RIPA buffer (Servicebio, Wuhan, China) to extract total protein. Total protein concentrations were determined using the BCA Protein Assay Kit (Cwbiotech, Beijing, China). A standardized western blotting protocol was adopted. Briefly, 10–20 μg of protein per channel was separated through 10% SDS-PAGE and then transferred to polyvinylidene difluoride (PVDF) membranes (Millipore, Billerica, MA, United States). After blocking with BSA (Roche, Basel, Switzerland) for 1 h, membranes were incubated with primary antibodies against 5-HT (ab10385, Abcam, Cambridge, United Kingdom, 1:1000), 5-HT_1A_ receptor (sc-10801, Santa Cruz, CA, United States, 1:1000), 5-HT_2C_ receptor (sc-10802, Santa Cruz, CA, United States, 1:1000), and SERT (sc-13997, Santa Cruz, CA, United States, 1:1000) at 4°C overnight. The membranes were washed, incubated with secondary antibody, and visualized using the DAB Kit (Servicebio, Wuhan, China) according to the manufacturer’s protocols. Specific bands were detected using a Gel Imaging System (Nikon, Japan). Gray values were quantified using Image-Pro Plus 6.0 (Media Cybernetics, United States) using β-actin as a control.

### Statistical Analyses

SPSS 19.0 statistical software (SPSS Inc., Chicago, IL, United States) was used for statistical analyses. The data are presented as mean ± SD and comparisons between groups were performed using one-way ANOVA. A result with *P* < 0.05 was considered statistically significant.

## Results

### Depression-Like Behaviors in EAP Rats

**FIGURE 1:**
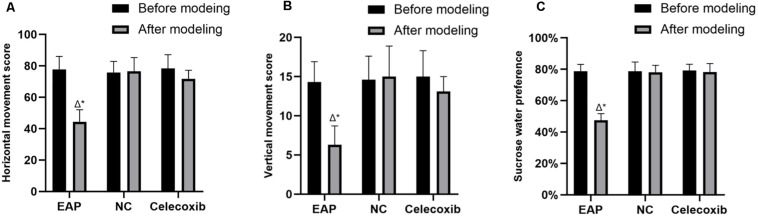
Results of the open-field test and sucrose consumption test for all three rat groups. Horizontal movement score **(A)**, vertical movement score **(B)**, and sucrose water preference **(C)** of EAP rats were reduced compared with the same rats before modeling or with the rats in the normal control and celecoxib groups. △*P* < 0.001 compared with the normal control group; *P < 0.001 compared with rats from the same group before establishment of the model; NC, Normal Control Group.

Behavioral changes were evaluated using the open-field test and the sucrose consumption test. EAP model rats exhibit typical depressive behaviors; thus, exploratory activity and sucrose water preference were reduced (Figure 1). These behaviors could be rescued by celecoxib treatment. Before model establishment, all rats exhibited normal active behaviors, and no significant difference in horizontal movement score, vertical movement score, or sucrose water preference was observed between the three groups (*P* > 0.05). However, 8 weeks after establishment of the EAP rat model, horizontal movement score (Figure 1A), vertical movement score (Figure 1B), and sucrose water preference (Figure 1C) were significantly reduced compared with the same rats before modeling (*P* < 0.001) or with the rats in the normal control and celecoxib groups (*P* < 0.001). No significant behavioral change was observed in the celecoxib or normal control groups during the study period (*P* > 0.05), and no difference was observed between the celecoxib group and the normal control group.

### Sexual Behavior Changes and Erectile Function of EAP Rats

**FIGURE 2:**
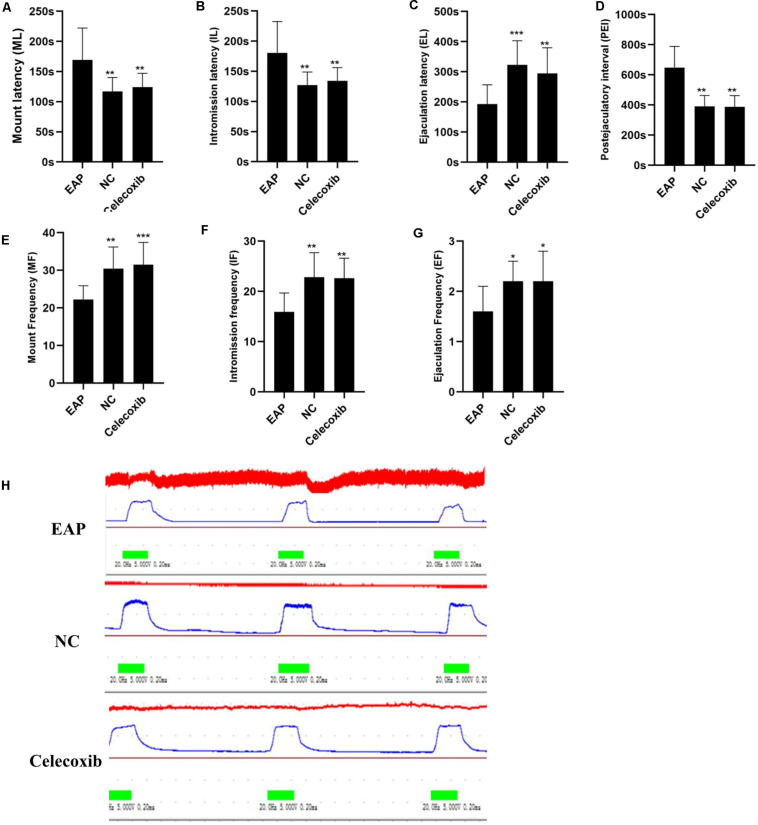
Autoimmune prostatitis impaired rat sexual function. Mount latency (ML) was increased in EAP rats compared with celecoxib group and normal control group rats **(A)**. In addition, intromission latency (IL) was elongated **(B)**, and Ejaculation latency (EL) and postejaculatory interval (PEI) were shortened **(C,D)** in EAP rats. Mount frequency (MF), intromission frequency (IF), and ejaculation frequency (EF) were all reduced **(E–G)**. No significant differences between the celecoxib group and the normal control group were observed. The representative ICP/MAP of the rats in all three groups were all above 0.70 **(H)**. *P < 0.05, **P < 0.01, ***P < 0.001 compared with EAP group.

The sexual behavior and erectile function of rats in all three groups were analyzed. While sexual behavior changes were observed in EAP rats when compared with the normal control group, no such changes were observed in the celecoxib group (Table 1 and Figures 2A–G). Mount frequency (MF), intromission frequency (IF), and ejaculation frequency (EF) were all significantly reduced in EAP rats compared with the celecoxib and normal control groups (*P* < 0.05) (Figures 2E–G), and mount latency (ML) was increased (*P* < 0.05) (Figure 2A). Moreover, intromission latency (IL) and postejaculatory interval (PEI) were increased in EAP rats (Figures 2B,D), while ejaculation latency (EL) was reduced (Figure 2C). No significant difference in any of the variables was observed between the celecoxib group and the normal control group (*P* > 0.05). The ICP/MAP were normal (above 0.70) in all three groups (Figure 2H), and no significant difference was observed between any two groups (*P* > 0.05).

**TABLE 1:** Sexual behavior parameters of rats in different groups.

**Parameters**	**EAP (*n* = 12)**	**NC (*n* = 10)**	**Celecoxib (*n* = 10)**	***F*-value**	***P*-value**
Mount latency (ML) (s)	169.3 ± 52.9	117.0 ± 23.0**	124.2 ± 22.9**	6.489	0.005
Intromission latency (IL) (s)	180.8 ± 51.8	127.2 ± 21.7**	134.1 ± 22.0**	7.272	0.003
ejaculation latency (EL) (s)	192.5 ± 64.5	323.0 ± 80.0***	294.2 ± 85.5**	9.016	0.001
Postejaculatory interval (PEI) (s)	648.3 ± 140.5	391.0 ± 71.8***	388.0 ± 73.8***	23.308	0
Mount frequency (MF)	22.2 ± 3.7	30.4 ± 5.8**	31.5 ± 5.9***	11.002	0
Intromission frequency (IF)	15.9 ± 3.8	22.8 ± 4.9**	22.6 ± 4.0**	9.649	0.001
Ejaculation frequency (EF)	1.6 ± 0.5	2.2 ± 0.4*	2.2 ± 0.6*	5.095	0.013

*P < 0.05, **P < 0.01, ***P < 0.001 compared with EAP group.

### Inflammatory Changes of Prostate and Increased Serum IL-1β in EAP Rats

**FIGURE 3:**
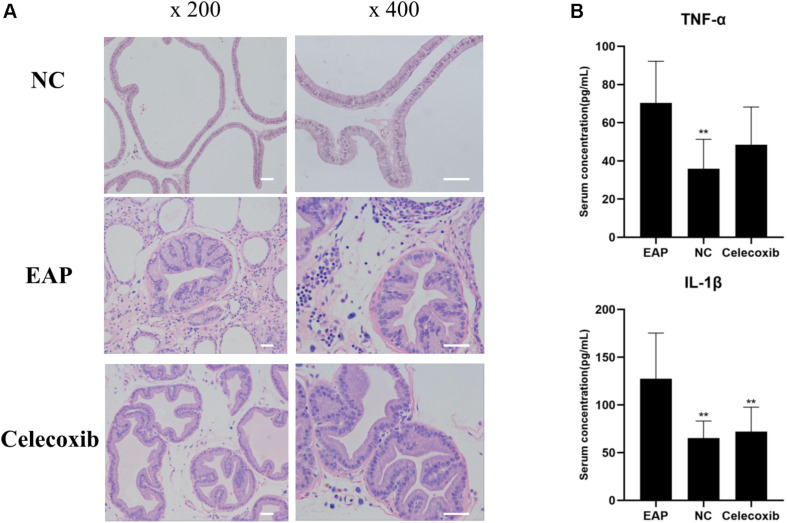
Inflammatory changes of prostate and increased serum IL-1β in EAP rats. Representative inflammatory changes in the prostate gland (HE staining) in EAP rats are shown **(A)**; TNF-α and IL-1β levels were increased in EAP group rats compared with the normal control (NC) group **(B)**. **P < 0.01 compared with EAP group. Scale bar = 25 μm.

To evaluate prostate and systemic inflammatory effects, histological examinations of rat prostate glands were performed and serum IL-1β and TNF-α concentrations were investigated. Characteristic inflammatory changes in the prostate gland were observed in 12 (out of 16) EAP group rats (Figure 3A). The lumens of these prostate glands were irregular in shape, secretions in the glandular cavity were scattered unevenly, the glandular epithelium showed segmental necrosis, and a large number of inflammatory cells had infiltrated into the interstitium. In the other 4 EAP group rats, HE staining showed normal morphology; this was adjudged to be due to the failure of modeling, and the data from these four rats were removed in all subsequent statistical analyses. In addition, serum IL-1β and TNF-α were both significantly increased compared with normal control group levels (*P* < 0.01) (Figure 3B). A comparison of celecoxib group and EAP group rats revealed that celecoxib treatment reduced infiltration of inflammatory cells and lowered serum IL-1β and TNF-α levels (Figure 3B); however, the observed reduction in TNF-α was not statistically significant. No significant differences were observed between celecoxib group and normal control group rats (*P* > 0.05) (Figure 3B).

### Expression of 5-HT System Regulators Was Altered in Hippocampus of EAP Rats

**FIGURE 4:**
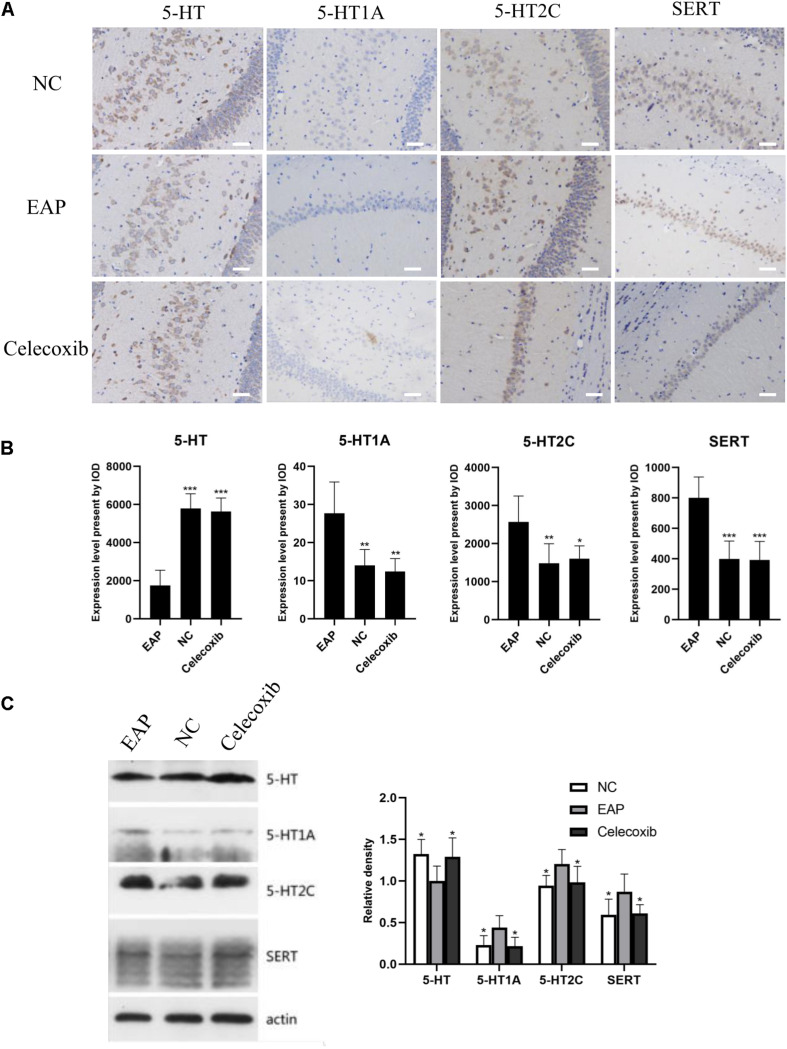
Expression levels of 5-HT, 5-HT_1A_, 5-HT_2C_ receptor, and SERT in rat hippocampus. In the EAP group (EAP), 5-HT was significantly decreased compared with the normal control group (NC) and celecoxib group (celecoxib), while 5-HT_1A_, 5-HT_2C_, and SERT were significantly increased **(B)**. Result were verified by IHC **(A)** and western blot **(C)**. *P < 0.05, **P < 0.01, ***P < 0.001 compared with EAP group. Scale bar = 25 μm.

To investigate changes in the 5-HT system in the hippocampus related to depression, the expression levels of 5-HT, 5-HT_1A_ receptor, 5-HT_2C_ receptor, and SERT in rat hippocampus were evaluated by immunohistochemistry and western blot analysis (Figure 4). In the EAP group, 5-HT expression in rat hippocampus was significantly decreased compared with the normal control group (*P* < 0.05) and celecoxib group (*P* < 0.05) rats (Figures 4B,C). In contrast, 5-HT_1A_, 5-HT_2C_, and SERT expression were all significantly increased (*P* < 0.05) (Figures 4B,C). No significant differences were observed between celecoxib group and normal control group rats. The western blot analyses were consistent with the results of quantitative analysis with IHC.

### Expression of 5-HT System Regulators Was Altered in Spinal Cord (T13-L1, L5–S2) of EAP Rats

**FIGURE 5:**
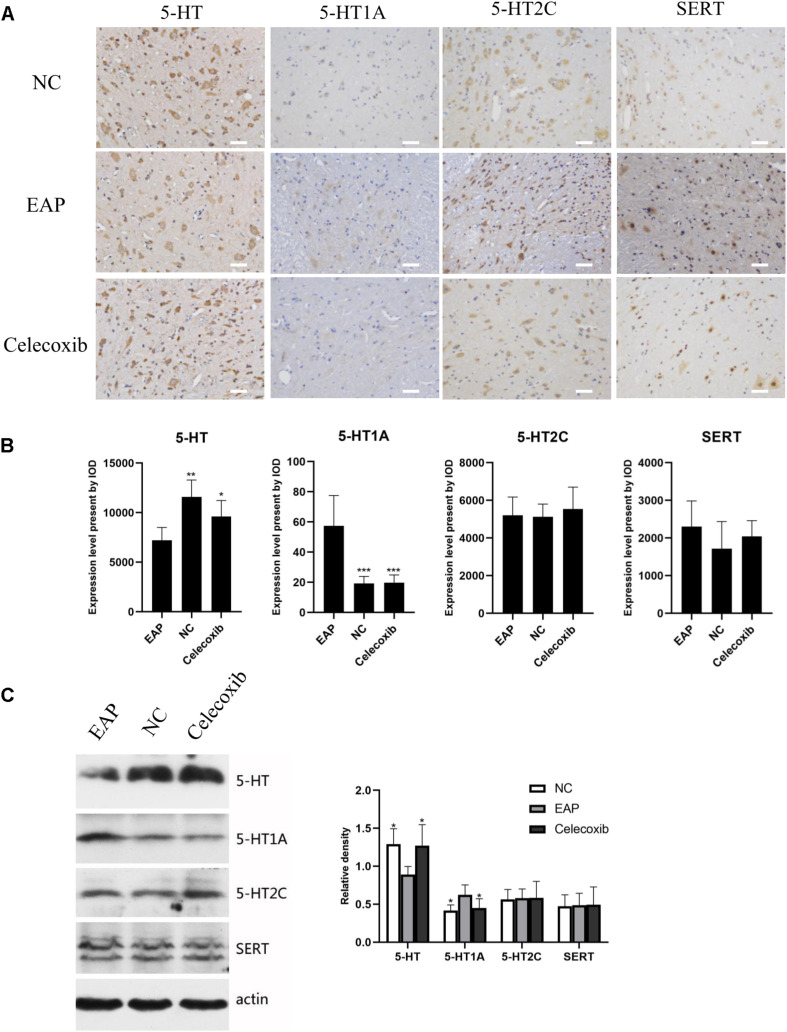
Expression levels of 5-HT, 5-HT_1A_, 5-HT_2C_ receptor, and SERT in rat T13–L1 segments of spinal cord. 5-HT levels were decreased and 5-HT_1A_ receptor levels were increased in the EAP group (EAP) compared with the normal control group (NC) and celecoxib group (celecoxib) **(B)**. Results were verified by IHC **(A)** and western blot **(C)**. *P < 0.05, **P < 0.01, ***P < 0.001 compared with EAP group. Scale bar = 25 μm.

**FIGURE 6:**
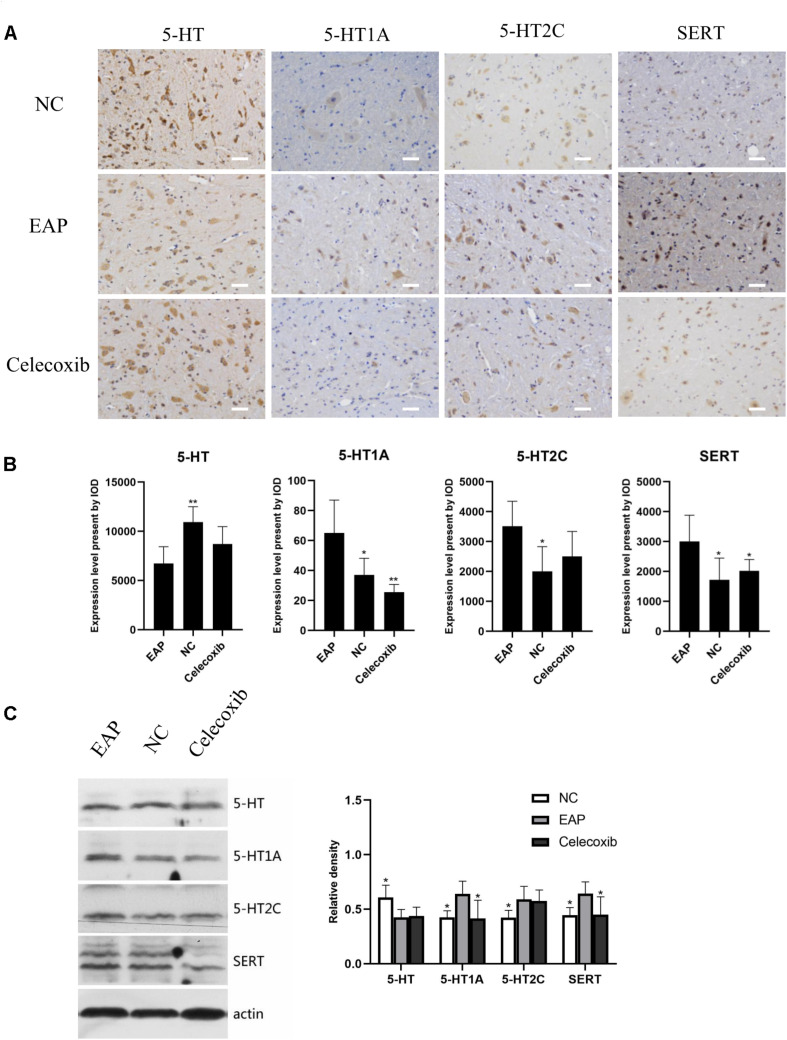
Expression levels of 5-HT, 5-HT_1A_, 5-HT_2C_ receptor, and SERT in rat L5–S2 segments of spinal cord. 5-HT levels were significantly decreased compared with normal control group (NC), while 5-HT_1A_, 5-HT_2C_, and SERT levels were significantly increased **(B)**. Result were verified by IHC **(A)** and western blot **(C)**. *P < 0.05, **P < 0.01, ***P < 0.001 compared with EAP group. Scale bar = 25 μm.

We also investigated changes in the 5-HT system in spinal segments controlling prostate sensation (Figures 5, 6). Expression of 5-HT was significantly decreased in T13–L1 spinal cord segments in EAP rats and expression of 5-HT_1A_ receptor was increased compared with normal control group (*P* < 0.05) and celecoxib group (*P* < 0.05) rats (Figures 5B,C). Again, expression levels in celecoxib group rats were restored to similar levels as observed in normal control group rats. In L5–S2 segments of the spinal cord, 5-HT expression decreased, while 5-HT_1A_, 5-HT_2C_, and SERT expression levels increased in the EAP group, compared with the normal control group (Figure 6). In the celecoxib group, HT_1A_ and SERT expression levels were reduced compared with the EAP group (Figures 6B,C). However, no significant differences in 5-HT and 5-HT_2C_ expression levels were observed between the celecoxib group and the EAP group. Furthermore, no significant differences were observed between the celecoxib group and normal control group rats. Similar results were obtained from IHC and western blot. The expression levels of serotonin regulators in the central nervous system of EAP rat are compared with those of normal control group rats in Table 2.

**TABLE 2:** Serotonin regulators changes in EAP group compared to normal control group.

**Regulators**	**Hippocampus**	**T13-L1**	**L5-S2**
5-HT	L	L	L
5-HT1A	H	H	H
5-HT2C	H	N	N
SERT	H	N	H

*L, lower than normal control group; H, higher than normal control group; N, No statistical significance.*

## Discussion

Chronic prostatitis/chronic pelvic pain syndrome is a multifactorial disease. Therefore, it is difficult to recapitulate all the characteristics of CP/CPPS in a single animal model. Animal models of CP/CPPS commonly used include the EAP model (14), spontaneous prostatitis model (24), hormone and castration induced model (25), urine reflux model (26), and the chemically induced model (27). In each of these models, a specific trigger causes inflammation in the prostate via a non-bacterial route. This commonality confirms the central role of inflammation during the process of CP/CPPS initiation and development. Evidence supporting the association of CP/CPPS with autoimmunity is increasing, and includes the detection of prostate antigen specific T lymphocytes in CP/CPPS patients (28). No obvious signs of infection were found in most of the CP/CPPS patients with increased semen leukocytes (29).

It may be concluded that EAP provides an ideal animal model of CP/CPPS. The EAP model is characterized by prostate specific inflammation, good stability, and long-term maintenance (30). Nonetheless, the success rate of EAP model establishment varies according to method and rodent animal used. In the present study, we constructed EAP model rats with a success rate of 75% (12/16). Although the success rate in establishing EAP mouse models can reach 100%, by using the rat model, intromission and ejaculation can be clearly observed. Additionally, the average ejaculation latencies of rat and human are more similar (about 5 min), although there are, of course, large individual differences both in human and rats (21). The characteristic inflammatory changes in the prostate demonstrated in our model provide a basis for the study of behavioral changes, sexual behavioral changes, and erectile function in our animal model of CP/CPPS, and for investigating the anti-inflammatory therapy efficacy of celecoxib.

The high risk of depression associated with CP/CPPS was confirmed in a clinical study and in EAP mice (6, 31). The depressive state of animals is mainly manifested by a decrease in overall activity, reduced social communication activities, reduced exploratory activities, and defects in aggression and pleasure sensation (23, 32). In our present study, the depressive behavior pattern observed in EAP rat was relieved by celecoxib treatment.

Sexual dysfunction is common in CP/CPPS patients. However, sexual dysfunction in the EAP rat model has not been previously confirmed. In the current study, we report impaired sexual function in EAP rats, confirming that this is an ideal model to study CP/CPPS related sexual dysfunction. In mating experiments, reduced MF, IF, and EF, and elongated ML, all reflect a decline in sexual drive. Moreover, a shortened EL implied a reduced ejaculation threshold (or a condition with premature ejaculation), while elongated IL and shortened PEI indicated increased threshold of erection (33). Following anti-inflammatory treatment in the celecoxib group, all these parameters of sexual behavior were rescued to normal levels. This result provides further evidence that autoimmune inflammation is a critical factor in the pathological mechanisms underlying CP/CPPS. To our surprise, the ICP/MAP under electrical stimulation of CN showed no significant change, which may indicate that no apparent organic lesion was caused in EAP rats. However, under an electrical stimulation condition, we were not able to assess the erectile function of rats in the natural state. And it should be noted that male rats as well as many other mammals have a baculum in the penis (34) which limit a rigorous assessment of erectile function.

Anti-inflammatory therapy using steroidal or non-steroidal anti-inflammatory drugs has been validated for the treatment of CP/CPPS. A meta-analysis has provided evidence that patients receiving anti-inflammatory treatment alone were 80% more likely to have favorable responses than patients receiving placebo. Moreover, α-blockers combined with anti-inflammatory treatment were associated with significantly better pain scores than placebo (18). In clinical practice, celecoxib has already been employed in the treatment of CP/CPPS, although the therapeutic effect faded 2 weeks after the end of treatment (17). The underlying mechanisms associated with prostate inflammation and anti-inflammation activity remain unclear. Several studies have shown that the proinflammatory factors TNF-α and IL-1β were increased in prostatic fluid, seminal plasma, or serum of CP/CPPS patients (35–37). Here, we detected significantly higher serum IL-1β and TNF-α levels in EAP rats compared with normal rats. In a comparison between the celecoxib and EAP groups, while IL-1β levels were significantly decreased in the celecoxib group, an observed decrease in TNF-α levels was not significant. These results demonstrate the anti-inflammatory efficacy of celecoxib and indicate that proinflammatory factors such as IL-1β are involved in CP/CPPS.

The serotonin system plays an important role in the occurrence of depression and male sexual dysfunction. However, studies researching the role of the serotonin system in sexual dysfunction are very limited. It has previously been suggested that low brain 5-HT levels (and 5-HT_1A_ receptor and 5-HT_2C_ receptor levels) were related to the pathogenesis of depression, though the conclusions are controversial (38). Increased serotonin levels in the central nervous system elevate the ejaculatory threshold (21, 22). However, 5-HT_1A_ receptors and 5-HT_2C_ receptors may play opposing biological roles in ejaculation. While 5-HT_1A_ receptors reduced ejaculation threshold, shortened ejaculation latency, and accelerated ejaculation, 5-HT_2C_ receptors delayed ejaculation (21). In addition, SERTs on the presynaptic membrane function by reabsorbing 5-HT, reducing the concentration of 5-HT in the synaptic gap, so as to increase ejaculation threshold and delay ejaculation (39). Based on these findings, we investigated the possible association between proinflammatory factors and the 5-HT system. Interestingly, the expression pattern of 5-HT system regulators was significantly altered in EAP rats. 5-HT expression levels were decreased and 5-HT_1A_ receptors expression levels were increased in the hippocampus and spinal cord (T13–L1 and L5–S2), and this may contribute to the depression-like behavior and PE. It is interesting to note that 5-HT_1A_ receptors are mainly distributed in the dorsal horn of the spinal cord and their expression can be upregulated by pain (40). Thus, pelvic pain in the EAP rat may attribute to the increased 5-HT_1A_ receptor expression in T13–L1 and L5–S2 segments, which are the segments of spinal cord controlling prostate sensation. Additionally, current evidence shows that depressive symptoms can be improved by administration of celecoxib in rats (19) as well as in humans (20), suggesting that celecoxib might relieve sexual dysfunction by psychological improvement exclusively, or combined with relief in prostate symptoms.

According to present understanding, proinflammatory cytokines can reduce 5-HT expression levels in the central nervous system in various ways (41): (i) an increase in proinflammatory cytokine levels leads to hyperactivity of the hypothalamic–pituitary–adrenal (HPA) axis, which stimulates the secretion of corticotropin releasing hormone (CRH) by paraventricular nuclei (PVN). CRH can reduce 5-HT release by acting on CRH receptors distributed in the limbic system (hippocampus, amygdala) and other parts of the brain; (ii) activation of indoleamine 2,3-dioxygenase (IDO) can be induced by NF-κB and p38 MAPK pathways. IDO can enhance the metabolism of tryptophan, a 5-HT precursor, and thus reduce the synthesis of 5-HT; (iii) the expression and activity of SERT can be up-regulated through the p38 MAPK pathway, which is activated by TNF-α and IL-1β. Uptake of 5-HT is promoted by SERT, and the level of 5-HT is lowered (42). Our findings support our preliminary hypotheses that inflammation can cause depression and sexual dysfunction via the serotonin system, and that this can be relieved by celecoxib via suppression of inflammatory factors. However, we acknowledge the limitations that the local levels of IL-1β and TNF-α in prostate were not assessed, and that other inflammatory factors related to CP/CPPS were not included in the present study. Moreover, the possible roles of the signal pathways mentioned above have not been elucidated. Therefore, more detailed studies are needed to understand the molecular mechanisms pertaining to inflammation and the 5-HT system.

In summary, our study provides a new insight into the mechanism of CP/CPPS in EAP rats. We demonstrate that autoimmune prostatitis can cause sexual function and depression in this rat model. And a serotonin system disorder in the central nervous system was likely mediated via inflammation in EAP rats. Moreover, we show that anti-inflammatory therapy with celecoxib was effective in relieving CP/CPPS characteristics in EAP rats.

## Data Availability Statement

The raw data supporting the conclusions of this article will be made available by the authors, without undue reservation.

## Ethics Statement

The animal study was reviewed and approved by the Ethics Committee of The First Affiliated Hospital of Sun Yat-sen University (Guangzhou, China).

## Author Contributions

All authors contributed to the intellectual content of the manuscript and approved the manuscript version submitted for publication. YaZ, XL, and KZ have contributed equally to this work. YaZ, KZ, and MZ performed the experiments. YaZ, XL, KX, and YX analyzed the data and prepared the figures. XL, KZ, YiZ, and YX interpreted the experimental results. YaZ and XL drafted the manuscript. CD and CC were responsible for the conception and design of the research and approved the final version of the manuscript. XS and CD edited and revised the manuscript.

## Conflict of Interest

The authors declare that the research was conducted in the absence of any commercial or financial relationships that could be construed as a potential conflict of interest.
